# Caffeine increases motor output entropy and performance in 4 km cycling time trial

**DOI:** 10.1371/journal.pone.0236592

**Published:** 2020-08-13

**Authors:** Bruno Ferreira Viana, Gabriel S. Trajano, Carlos Ugrinowitsch, Flávio Oliveira Pires

**Affiliations:** 1 Physical Education course, Augusto Motta University Center (UNISUAM), Rio de Janeiro, RJ, Brazil; 2 Physical Education course, Estácio de Sá University (UNESA), Rio de Janeiro, RJ, Brazil; 3 Exercise Psychophysiology Research Group, School of Arts, Sciences and Humanities, University of São Paulo, SP, Brazil; 4 School of Exercise and Nutrition Sciences, Queensland University of Technology, Kelvin Grove, QLD, Australia; 5 Institute of Health and Biomedical Innovation, Queensland University of Technology, Kelvin Grove, QLD, Australia; 6 School of Physical Education and Sport, University of São Paulo, SP, Brazil; Mary Baldwin University Murphy Deming College of Health Sciences, UNITED STATES

## Abstract

Caffeine improves cycling time trial performance through enhanced motor output and muscle recruitment. However, it is unknown if caffeine further increases power output entropy. To investigate the effects of caffeine effects on cycling time trial performance and motor output entropy (MOEn), nine cyclists (VO_2MAX_ of 55 ± 6.1 mL^.^kg^.-1^min^-1^) performed a 4 km cycling time trial (TT_4km_) after caffeine and placebo ingestion in a counterbalanced order. Power output data were sampled at a 2 Hz frequency, thereafter entropy was estimated on a sliding-window fashion to generate a power output time series. A number of mixed models compared performance and motor output entropy between caffeine and placebo every 25% of the total TT_4km_ distance. Caffeine ingestion improved power output by 8% (p = 0.003) and increased MOEn by 7% (p = 0.018). Cyclists adopted a U-shaped pacing strategy after caffeine ingestion. MOEn mirrored power output responses as an inverted U-shape MOEn during the time trial. Accordingly, a strong inverse correlation was observed between MOEn and power output responses over the last 25% of the TT_4km_ (p < 0.001), regardless of the ingestion, likely reflecting the end spurt during this period (p = 0.016). Caffeine ingestion improved TT_4km_ performance and motor output responses likely due to a greater power output entropy.

## Introduction

According to the dynamic system theory, the variability presented by a given physiological system, a concept that is known as complexity, may reflect its flexibility to face natural perturbations [1,2]. For example, the neuromuscular system is characterized by regular fluctuations in electrophysiological responses (i.e. complexity) which enable the central nervous system (CNS) to adapt to environment-induced perturbations [3]. Assuming that every single body motion is a dynamic acceleration-deceleration interplay [4], the level of complexity in motor output responses may indicate the CNS ability to face a physical task-induced perturbation. Studies have reported an association between motor output complexity and fatigue, as a reduced knee extensor torque entropy has been observed as a fatiguing single-joint isometric exercise progresses [5,6]. In this regard, it has been hypothesized that a “loss of complexity” is likely present in fatiguing exercises, so that variations in neuromuscular complexity such as in motor output entropy (MOEn), may indicate the neuromuscular system ability to face the exercise-induced fatigue [7].

Studies investigating the MOEn-fatigue relationship have used highly controlled isometric muscle tasks as an exercise mode [5,6]. Despite providing a well-controlled intensity and power output response, this exercise mode has a low ecological validity as it reflects an unnatural form of exercise. Consequently, isometric muscle task-derived results cannot provide enough to understand the MOEn in more usual forms of exercise. For example, exercises such as a cycling time trial may be insightful to understand the MOEn-fatigue relationship in strenuous whole-body self-paced exercises, as this exercise mode requires a more complex, moment-to-moment regulation when regulating pacing and exercise performance [8–15]. For example, power output fluctuations during a cycling time trial may indicate the CNS ability to deal with the central-peripheral fatigue interplay during a more natural form of exercise [8,15], thereby offering insights on the role of the neuromuscular complexity in exercise regulation and pacing strategy.

Whether both central and peripheral fatigue increase as a cycling time trial progresses, one may argue that the neuromuscular complexity decreases as a function of the trial distance [16]. Consequently, a likely U-shape pacing strategy during a short cycling trial [17] may indicate a reduction in MOEn, what could be related to the trial fatigue status. Importantly, a shorter cycling time trial may be preferable to emphasize the CNS complexity when regulating the motor output during exercise, given that the magnitude of neural drive required to complete a short time trial such as 4km (TT_4km_) is greater than the neural drive necessary to complete longer ones (e.g. 40km). In this sense, a higher power output could suggest an enhanced motor unit firing synchronization during exercise, as the electromyography (EMG) entropy is lower in higher (i.e. 330 W) than lower (i.e. 150 W) power output values [18]. Therefore, considering that trained cyclists produce a higher mean power output in short (TT_4km_) than in long cycling time trials (i.e. 40km) [16], analysis of MOEn in TT_4km_ could elucidate the MOEn-fatigue interplay in a high ecological validity exercise.

Some ergogenic aids could add valuable information to the neuromuscular complexity-cycling paradigm, as some ergogenics have the ability to change neuromuscular properties. For example, caffeine may be an interesting approach to investigate the MOEn-fatigue interplay, indicating if fluctuations in power output responses during cycling time trials may be related to changes in neuromuscular response complexity. It has been suggested that caffeine increases MOEn through amplification of the synaptic inputs to α-motor neurons [19]. Caffeine increases the monoamines synthesis and turnover [20], thereby amplifying the synaptic input and motoneuronal gain [21,22] as indicated by a steeper H-reflex curve and greater self-sustained motor unit firing frequency [22,23]. Consequently, assuming that a higher motor neuron gain is associated with a greater muscle force variability as suggested elsewhere [24], one may hypothesize that caffeine increases MOEn through increased neuromuscular complexity. Assuming this hypothesis is right, one may also expect that caffeine may further attenuate the fatigue-induced reduction in MOEn as the trial progresses, thereby likely improving power output and performance [25].

Therefore, the present study aimed to characterize MOEn in a TT_4km_ and verify if caffeine ingestion increases power output complexity and performance in this trial. We hypothesized that caffeine would attenuate a fatigue-induced reduction in power output complexity, improving power output and performance during TT_4km_.

## Methods

### Participants and experimental design

Nine endurance-trained male cyclists (32.0 ± 7.5 years, body mass of 74.9 ± 8.6 kg, height of 1.73 ± 5.2 m, VO_2MAX_ of 55.0 ± 6.1 mL^.^kg^-1.^min^-1^,) having a minimum 3 years training experience competing at regional competitions, classified as performance level 3 [26] and experienced in cycling time trials, volunteered to participate in this study. They were non-smokers and had no neuromuscular or cardiopulmonary disorder that could affect the study outcomes. Most cyclists (n = 7) were low-to-moderate consumers of caffeine (50–250 mg of caffeine per day) and two were classified as non-consumers (≤50 mg of caffeine per day), according to classification used elsewhere [27,28]. The experimental procedures were previously approved by the Research Ethics Committee of the University of São Paulo (#0023.0.342.000–10) and explained to participants before the informed consent form signature.

After a preliminary visit to obtain anthropometric measures and assess the VO_2MAX_ through a maximal incremental cycling exercise performed with a 80 rpm pedal cadence (25 W·min^-1^ increases until exhaustion), cyclists attended to 3 sessions in a counterbalanced order; 1) a baseline 4 km cycling time trial (TT_4km_); 2) a TT_4km_ after caffeine ingestion; 3) a TT_4km_ after placebo ingestion. All visits were interspersed by a ~7 days interval. The cyclists were encouraged to maintain the training schedule (intensity and volume) throughout the study period and avoid vigorous exercise, alcohol, and stimulant or caffeine beverages for the last 24 h before the sessions. Briefly, we chose a TT_4km_ as a strenuous whole-body self-paced exercise and assumed that endurance-trained cyclists complete this trial having a mean power output higher than 300 W [15,16], therefore potentiating a likely reduction in MOEn [18]. In contrast, caffeine ingestion may increase MOEn and TT_4km_ performance.

### Caffeine and placebo ingestion

Caffeine and placebo capsules (6 mg^.^kg^-1^ of body mass) were ingested ~ 60 min before the TT_4km_ commencement. Caffeine and sucrose-based (i.e. placebo) substances were formulated in opaque capsules of equal size, color and taste to prevent that participants rightly guessed the treatment. Importantly, instead of a double-blind, randomized placebo-controlled clinical trial, we used a placebo-deceived design, as some have argued that the use of double-blind designs is a possible source of bias in clinical trials [29,30]. To ensure that eventual differences between caffeine and placebo were solely due to caffeine pharmacological effects, cyclists were led to believe they ingested caffeine in both sessions and the study was investigating the reproducibility of caffeine effects on TT_4km_ performance. They were informed about the presence of a placebo condition at the study completion, as reported elsewhere [31]. Informal and anecdotal communication revealed that participants were blinded about the presence of a true placebo pill.

### Instruments, measures, and analysis

All cyclists performed the TT_4km_ on the same road bike (Giant®, Thousand Oaks, CA, USA) attached to a cycle-simulator calibrated before every test (Racer Mate®, Computrainer, Seattle, WA, EUA), individually fitted with crank, pedals and saddle. This equipment provided power output measures (W) at a 2Hz sampling rate. The validity and reliability of this system have been previously reported [32,33]. Cyclists performed a standard 7 min warm-up, consisting of a 5 min self-paced (gear and cadence freely adjusted) and a 2 min controlled-pace cycling (fixed gear at 100 W and 80 rpm pedal cadence). When they were still cycling at the end of the controlled-pace warmup, they immediately started the TT_4km_. The cyclists were oriented to rate their perceived exertion (RPE) at each 0.5 km, according to the 6–20 Borg’s scale [34], so that the mean RPE during the TT_4km_ was calculated. A researcher unaware of the substance ingested encouraged the cyclists to complete the distance as fast as possible, while distance feedback was available to cyclists to pace themselves.

### Entropy calculation

The entropy could be interpreted as a non-linear analysis that provides a measure of the complexity of a system [35]. Based on the information theory, entropy is a measure that reflects the level of uncertainty of a dataset or time series. Entropy can be obtained as the probability (*p_k_*) of each possible event multiplied by log of the inverse probability of each event ($\log\left(\frac{1}{p_{k}}\right)$) [36] as described in Eq 1.

$$H=\sum_{i=1}^{N}p_{k}\log\left(\frac{1}{p_{k}}\right)$$ (1)

However, the prior knowledge of the probability (*p_k_*) for the occurrence of all events is impossible in stochastic processes, therefore, adequate methodologies such as the sample entropy (SampEn) have been suggested [37]. The SampEn (Eq 2) fits the approximate entropy [38] to generate less time series length-dependence and self-matching-reduced bias (Eq 2).

$$SampEn(m,r,N)=-\ln\left(\frac{A_{m+1}(r)}{A_{m}(r)}\right)$$ (2)

Where *m* is the length of sequences to be compared, *r* is the tolerance for accepting matches and *N* is the length of the time series. In the present study, the input parameters were set as *r* = 0.2, *m* = 2, *N* = 120. In the SampEn algorithm, *r* is multiplied by the standard deviation (SD) of *N*, providing a matching threshold and allowing comparisons among sequences of *m* points. Readers are referred to a seminal work by Richman et al. [37] for a comprehensive SampEn demonstration.

### Data analysis and statistics

In this study MOEn was estimated applying SampEn algorithm in the mechanical power output signal obtained during TT_4km_. A custom code (Matlab v.2013a, The Mathworks, EUA) was used to estimate MOEn over time, by applying a sliding-window over 120 samples epochs having 10 samples overlap. Thereafter, absolute power output data, as well as MOEn vectors, were expressed at each 25% of the total TT_4km_ distance (i.e. 25%, 50%, 75% and 100% of the trial).

Data were reported as mean (± SD) and 95% confidence limits (CI 95%). Power output and MOEn obtained at each 25% of the cycling trial were compared through a number of mixed models, having substance (caffeine and placebo) and distance (25%, 50%, 75% and 100% of the TT_4km_) as fixed factors, and cyclists as the random factor. The Pearson correlation coefficient was calculated between mean values of power output and MOEn for each 25% of the TT_4km_, as we expected that MOEn would decrease if cyclists significantly increased the power output. Significant results were accepted as p < 0.05 (SPSS software, version 17.0, SPSS Inc., Chicago, IL, USA).

## Results

**Fig 1:**
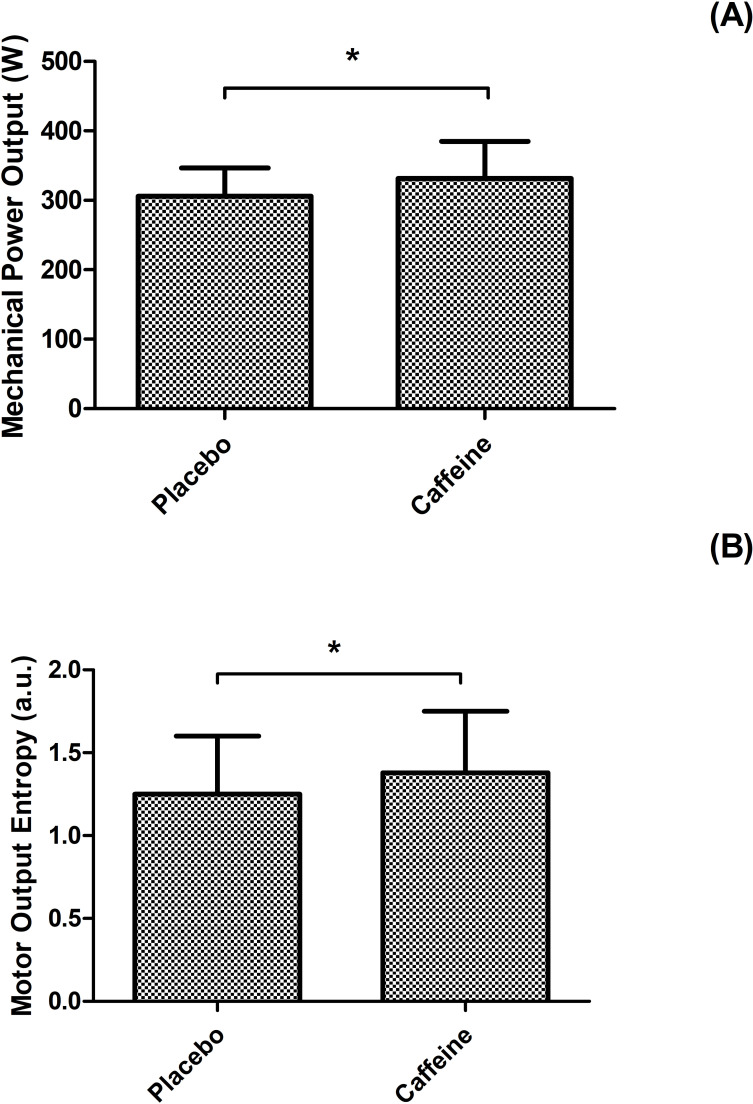
Cycling time trial performance and entropy. Mechanical power output (panel A) and motor output entropy (panel B) in placebo and caffeine trials. * indicates supplementation main effect in power output (p = 0.003, F = 9.69) and motor output entropy (p = 0.018, F = 5.983).

Ingestion of caffeine resulted in a 8% increase in mean power output (p = 0.003, F = 9.69) when compared to placebo, as mean power output was 331.4 ± 53 W (CI 95% [306.5–356.3]) in caffeine vs 306.2 ± 40 W (CI 95% [281.3–331.1]) in placebo (Fig 1A). This improved power output in caffeine was reflected in ~1.8% shorter times (p > 0.05) in caffeine (350.0 ± 14.6 s) than placebo (357.0 ± 13.2 s). Additionally, cyclists presented comparable mean RPE during the TT_4km_ in both supplementations with caffeine (16 ± 0.62 a.u.) and placebo (16 ± 0.63 a.u.).

**Fig 2:**
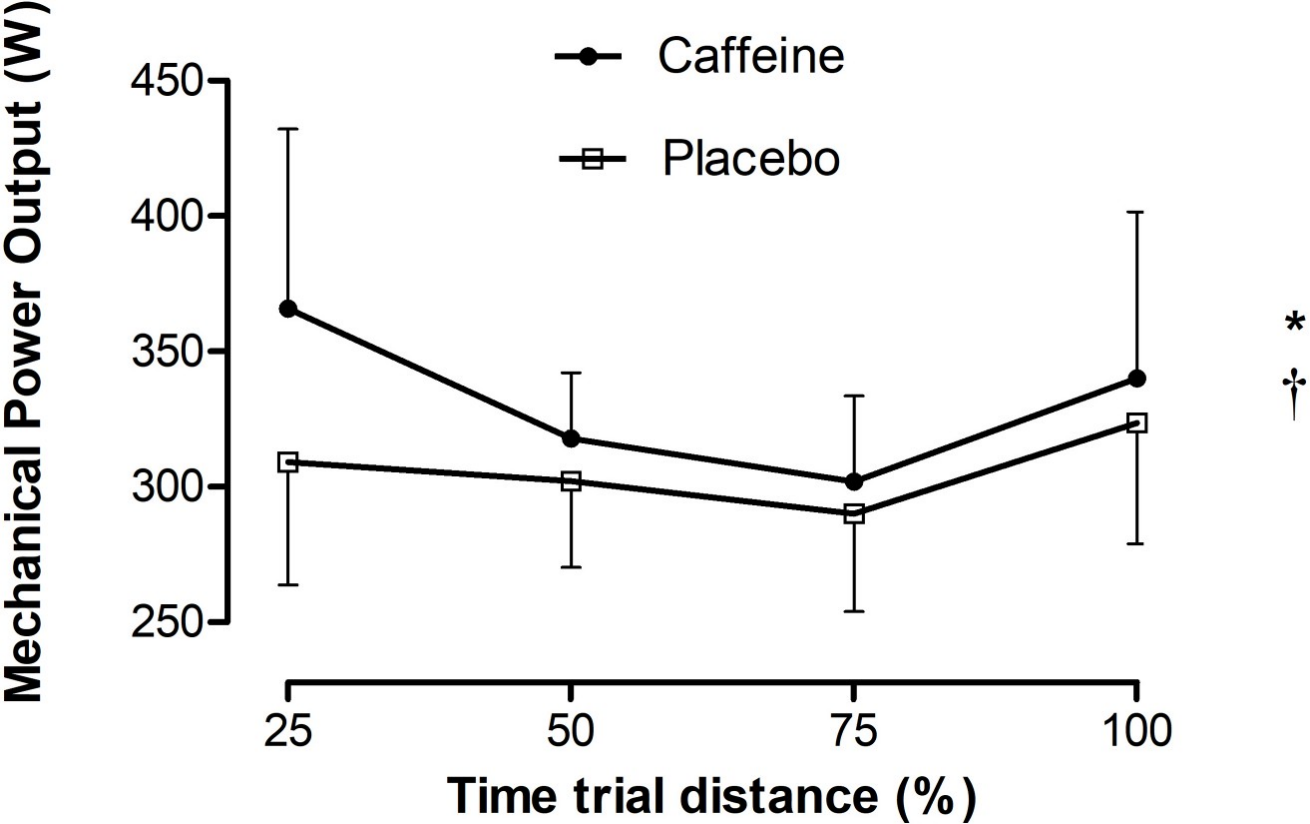
Pacing strategy. Mechanical power output relative to a percentage of the TT_4km_ distance. * indicates distance main effect (p = 0.002, F = 5.70) and † indicates supplementation main effect (p = 0.003, F = 9.69).

Cyclists adopted a U-shaped pacing strategy (Fig 2) so that a distance main effect was detected (p = 0.002, F = 5.70), and power output decreased by 14% from 25% to 75% of the TT_4km_ (-41.6 ± 11.4 w; CI 95% [-10.3, -72.9], p = 0.004), but increased by 11% from 75% to 100% of the TT_4km_ (35.9 ± 11.4 w; CI 95% [4.6, 67.2], p = 0.016). No substance by distance interaction effects were found in power output responses (p = 0.178, F = 1.697).

**Fig 3:**
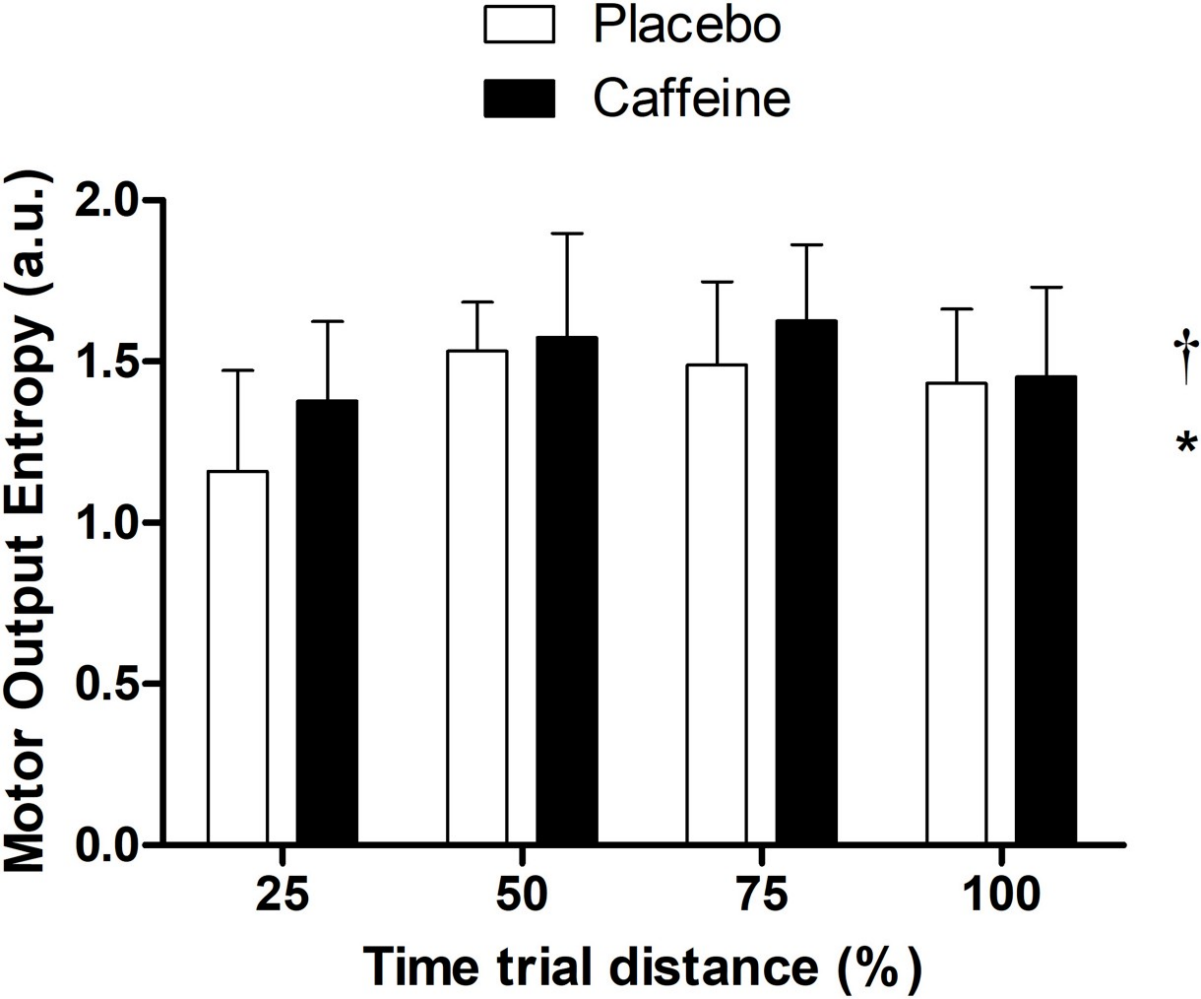
Motor output entropy during the cycling time trial. Motor output entropy was expressed relative to a percentage of the TT_4km_ distance. * indicates distance main effect (p < 0.001, F = 10.11) and † indicates supplementation main effect (p = 0.018, F = 5.98).

We observed a substance main effect on MOEn results, as MOEn was 7% greater in caffeine than placebo (p = 0.018, F = 5.983; CI 95% [0.019, 0.190]) (Fig 1B). We observed a distance main effect in MOEn (p < 0.001, F = 10,118; CI 95% [0.032, 0.339]), so that MOEn increased by ~ 20% from 25% to 50% of the trial (0.284 ± 0.060 A.U.; CI 95% [0.120, -0.449], p < 0.001), but remained unchanged between 50% and 75% (- 0.005 ± 0.06 A.U.; CI 95% [- 0.170, 0.160], p < 0.05) and between 75% and 100% (- 0.115 ± 0.060 A.U.; CI 95% [- 0.280, 0.050], p = 0.368). No substance by distance interaction effect was found in MOEn (p = 0.337, F = 1.151). Fig 3 depicts MOEn responses during the cycling trial, and Table 1 shows individual power output and MOEn responses over the TT_4km_ in both supplementations.

**Table 1:** Individual power output (PO) and motor output entropy (MOEn) responses were reported as a percentage of the total cycling time trial distance.

		Time trial distance (%)							
		25		50		75		100	
**Cyclists**	**Caffeine**	**PO**	**MOEn**	**PO**	**MOEn**	**PO**	**MOEn**	**PO**	**MOEn**
1	** **	342.34	1.19	290.04	1.31	270.39	1.56	288.26	1.31
2	** **	315.38	1.19	344.43	1.1	301.1	1.64	324.77	1.37
3	** **	336.24	1.62	322.99	1.95	309.11	1.84	308.23	1.68
4	** **	305.98	1.39	243.81	2.08	234.07	2.03	238.69	1.96
5	** **	379.99	1.11	299.51	1.45	274.32	1.31	262.33	1.39
6	** **	322.42	1.37	339.16	1.45	366.02	1.32	386.87	1.49
7	** **	376.9	1.13	311.7	1.46	299.67	1.53	302.76	1.03
8	** **	341.9	1.78	300.49	1.88	280.63	1.78	246.09	1.64
9	** **	344.6	1.62	326.72	1.48	335.18	1.61	364.72	1.35
	**Placebo**	**PO**	**MOEn**	**PO**	**MOEn**	**PO**	**MOEn**	**PO**	**MOEn**
1	** **	308.22	1.21	291.75	1.48	267.53	1.32	268.5	1.63
2	** **	298.72	1.24	339.59	1.44	319.98	0.91	342.24	1.03
3	** **	349.09	1.19	283.82	1.7	268.22	1.67	256.75	1.61
4	** **	253.83	1.28	235.22	1.75	224.6	1.69	240.1	1.68
5	** **	365.88	0.58	297.12	1.38	275.27	1.59	291.38	1.36
6	** **	364.19	0.9	370.11	1.38	359.13	1.51	364.02	1.38
7	** **	369.75	0.99	295.63	1.57	281.31	1.5	287.63	1.55
8	** **	311.61	1.7	308	1.7	310.87	1.77	320.73	1.54
9	** **	365.57	1.35	311.37	1.39	314.31	1.45	370.08	1.12

Correlations analysis revealed that MOEn was inversely correlated with power output in the first 25% (r = - 0.82; p < 0.001) of placebo condition, but not in caffeine. Negative correlations were also founded between 25% and 50% of the TT_4km_ in caffeine (r = - 0.76; p = 0.03), but not in placebo, perhaps as a result of the steady power distribution in placebo during this part of the trial. Furthermore, MOEn was inversely correlated with power output in the last 25% of the TT_4km_ in both caffeine (r = - 0.92, p < 0.001) and placebo trials (r = - 0.83, p < 0.001), being coincident with a ~11% increase in power output at the end of the trial, regardless of the supplementation. Table 2 shows all correlation coefficients between MOEn and power output.

**Table 2:** Pearson’s correlation coefficient between power outptut (PO) and motor output entropy (MOEn) over the 4km cycling time trial (TT4km) expressed as a percentage of the trial distance, in both caffeine and placebo supplementations.

%TT_4km_	Caffeine	p-value	Placebo	p-value
**0–25**	-0.25	0.33	-0.82	< 0.001
**25–50**	-0.76	0.03	-0.36	0.15
**50–75**	-0.36	0.16	-0.30	0.25
**75–100**	-0.92	< 0.001	-0.83	< 0.001

## Discussion

This study aimed to characterize the MOEn during a TT_4km_ and investigate if caffeine could change the MOEn-fatigue interplay during this strenuous, whole-body short cycling exercise. Our results showed a progressive reduction in motor output complexity as the TT_4km_ progressed, however caffeine increased TT_4km_ performance through an altered MOEn-fatigue interplay. These results may support the notion that caffeine increases power output responses and attenuates the fatigue-induced reduction in MOEn during TT_4km_.

This is the first study characterizing the MOEn during a natural exercise mode with high ecological validity such as a strenuous, whole-body short cycling time trial. In the present study, cyclists used a U-shaped pacing strategy to complete the TT_4km_, as they yielded an end spurt in the last 25% of the trial, after an increased power output in the initial 25% and unaltered power output in the intermediate 50%. In contrast, there was a progressive reduction in MOEn in the last 25% of the TT_4km_, regardless of the ingested substance, thereby supporting the fatigue-induced loss of entropy hypothesis as suggested in single-joint isometric exercises [6,39]. Briefly, MOEn responses could involve changes in neuromuscular complexity such as in CNS areas such as cortical, subcortical and spinal areas, as well as in motor neuron conduction to skeletal muscles. In this regard, the 20% increase in SampEn during the first half of the TT_4km_ was likely due to an enhanced exercise-induced perturbation, given that most relevant increases in psychophysiological responses take place in this part of the trial [15]. However, despite the increasing exercise-induced perturbation, neuromuscular fatigue was likely low over this half of the trial and probably allowed an increased MOEn when regulating the motor output during this part [40].

In the present study, we observed that cyclists attacked the first 25% of the TT_4km_ more aggressively when they ingested caffeine rather than placebo, somehow influencing the significant inverse correlation between power output and MOEn observed only with caffeine for this part of the trial. One may argue that neuromuscular fatigue is low during this initial part of the trial, thus likely allowing an adequate response of the neuromuscular system to the exercise-imposed perturbation through an increased motor unit firing variability. Moreover, the power output reduction observed from 25% to 50% of the caffeine TT_4km_ resulted in an inverse correlation between MOEn and power output during this part of the trial. In contrast, such a correlation between power output and MOEn was not observed in placebo TT_4km_ during these parts. In particular, the lowest MOEn and power output values were observed from 50% to 75% of the trials, so that no correlation between MOEn and power output was observed during this part, regardless of the ingested substance. Importantly, MOEn was inversely correlated with power output during the last 25% of the TT_4km_, regardless the ingested substance. This is a part of the cycling trial usually characterized by a sharp increase in power output (i.e. end spurt), so that one may hypothesize that the loss of MOEn during this latter part of the TT_4km_ was possibly related to a higher motor unit firing frequency, as neuromuscular fatigue is higher in the second half of a cycling trial [40].

A short cycling time trial having an end spurt may be a challenging scenario for the neuromuscular system, as this may represent fewer chances to vary muscle recruitment during pedaling mainly at the final stages of the trial [18], thereby reducing the mechanical power output variability (i.e. power output bandwidth) and MOEn. This hypothesis is based on a previous study that reported a different neuromuscular strategy as indicated by EMG analysis when contrasting fixed-load cycling at 150 W vs 300 W [18]. The authors of that study concluded that the lower EMG entropy observed during higher cycling power output was likely due to a higher synchronism of motor units firing.

The present study hypothesized that caffeine may increase MOEn by increasing motoneuronal gain and changing the input-output relationship in the motor pathway, thereby resulting in a greater variability in motor output. Although caffeine effects on skeletal muscles cannot be ruled out [41], the most convincing caffeine mechanism involves its action on neuronal A_1_ adenosine receptors, as improvements in exercise performance after caffeine ingestion have been associated with increases in spinal and supraspinal excitability [42,43]. Accordingly, the 7% increase observed in MOEn during the TT_4km_ after caffeine ingestion may be related to the caffeine’s action on neuronal tissue. Considering the 8% increase in mean power output in caffeine, one may argue that the higher power output observed in this condition was also related to a higher synchronism of motor units firing [18].

Analysis of movement variability have been used in different research fields [1,2,44], so that such analysis have been recently incorporated in neuromuscular fatigue studies [5,6]. In an exercise performance scenario, nonlinear measures such as MOEn may be a useful mean to estimate exercise-induced neuromuscular fatigue and its repercussion on motor control and performance responses [5]. Therefore, such a nonlinear measure could be helpful to improve the understanding of exercise performance and fatigue in different fields of sports sciences.

### Limitations and methodological considerations

The increased motoneuronal gain suggestion should be interpreted with caution, as no specific measures were performed to indicate motoneuronal gain. Insights to a motoneuronal gain mechanism could be obtained with advanced EMG techniques, such as the motor unit decomposition algorithms from electrode matrices-derived signal [45]. However, this technique is still restricted to low-intensity isometric contractions so that the dynamic whole-body exercise used in the present study limited the use of these measures to provide motoneuronal gain mechanisms insights after caffeine ingestion. Future studies comparing recruitment and de-recruitment frequencies of pairs of motor units could shed-light on caffeine effects on motoneuronal gain during voluntary contractions [46].

The present study is descriptive rather than mechanistic, and its design and methods may not elucidate if losses in power output entropy during cycling time trial were due to central or peripheral fatigue factors. In this sense, the power output was sampled at a 2 Hz frequency, a sampling rate that may not detect all variability in power output data, given the possible aliasing effect resulted from sampling the data in different pedal positions at each revolution. Another limitation was the absence of EMG responses, a measure that could have assessed the neuromuscular system and power output entropy, simultaneously.

Furthermore, we disregarded eventual subgroup comparisons based on the habitual caffeine consumption effects on performance, given that a recent well-designed study [28] and an important sports nutrients position stand challenged [27] the myth that habituation to caffeine consumption affects the caffeine’s potential as an ergogenic aid. However, considering that habitual caffeine consumption may change physiological responses to caffeine supplementation such as heart rate and ventilation, future studies may want to investigate potential habitual caffeine consumption effects on MOEn and EMG during cycling time trial.

## Conclusion

Results of the present study showed a progressive reduction in MOEn during the TT_4km_, thus revealing a progressive loss of motor output complexity as the trial progressed, mainly during the last 25% of the TT_4km_. However, caffeine ingestion improved TT_4km_ performance and MOEn. These results reinforce a likely fatigue-induced loss of complexity hypothesis.

## Supporting information

S1 Raw data (XLSX)

## References

[1] Lipsitz L. A. and Goldberger A. L. Loss of; complexity; and aging: Potential applications of fractals and chaos theory to senescence. JAMA 1992; 267(13): 1806–1809 10.1001/jama.1992.03480130122036 1482430

[2] Pincus S. M. Assessing Serial Irregularity and Its Implications for Health. Annals of the New York Academy of Sciences 2001; 954(1): 245–267 10.1111/j.1749-6632.2001.tb02755.x 11797860

[3] Hogan M. J., O’Hora D., Kiefer M., Kubesch S., Kilmartin L., Collins P., et al The effects of cardiorespiratory fitness and acute aerobic exercise on executive functioning and EEG entropy in adolescents. Frontiers in Human Neuroscience 2015; 9(538) 10.3389/fnhum.2015.00538 26539093 PMC4609754

[4] Merfeld D. M., Zupan L. and Peterka R. J. Humans use internal models to estimate gravity and linear acceleration. Nature 1999; 398(6728): 615–618 10.1038/19303 10217143

[5] Pethick J., Winter S. L. and Burnley M. Fatigue reduces the complexity of knee extensor torque fluctuations during maximal and submaximal intermittent isometric contractions in man. The Journal of Physiology 2015; 93(8): 2085–2096 10.1113/jphysiol.2015.284380 25664928 PMC4405761

[6] Pethick J., Winter S. L. and Burnley M. Loss of knee extensor torque complexity during fatiguing isometric muscle contractions occurs exclusively above the critical torque." American Journal of Physiology—Regulatory, Integrative and Comparative Physiology 2016; 310(11): R1144–R1153 10.1152/ajpregu.00019.2016 27101290

[7] Hong S. L. and Newell K. M. Entropy compensation in human motor adaptation. Chaos 2008 18(1): 013108 10.1063/1.2838854 18377059

[8] St Clair Gibson A. and Noakes T. D. Evidence for complex system integration and dynamic neural regulation of skeletal muscle recruitment during exercise in humans. Br J Sports Med 2004; 38(6): 797–806 10.1136/bjsm.2003.009852 15562183 PMC1724966

[9] St Clair Gibson A., Lambert E. V., Rauch L. H., Tucker R., Baden D. A., Foster C., et al The role of information processing between the brain and peripheral physiological systems in pacing and perception of effort. Sports Med 2006; 36(8): 705–722 10.2165/00007256-200636080-00006 16869711

[10] Tucker R., Bester A., Lambert E. V., Noakes T. D., Vaughan C. L. and St Clair Gibson A. Non-random fluctuations in power output during self-paced exercise. Br J Sports Med 2006; 40(11): 912–917; discussion 917 10.1136/bjsm.2006.026435 16980537 PMC2465046

[11] Marino F. The limitations of the constant load and self-paced exercise models of exercise physiology. Comparative Exercise Physiology 2010; 7(4): 6 10.1017/S1755254012000013

[12] Angus S. D. and Waterhouse B. J. Pacing strategy from high-frequency field data: more evidence for neural regulation? Med Sci Sports Exerc 2011; 43(12): 2405–2411 10.1249/MSS.0b013e3182245367 21606868

[13] Marino F. E., Gard M. and Drinkwater E. J. The limits to exercise performance and the future of fatigue research. Br J Sports Med 2011; 45(1): 65–67 10.1136/bjsm.2009.067611 19955162

[14] Smits B. L. M., Pepping G.-J. and Hettinga F. J. Pacing and Decision Making in Sport and Exercise: The Roles of Perception and Action in the Regulation of Exercise Intensity. Sports Medicine 2014; 44(6): 763–775 10.1007/s40279-014-0163-0 24706362

[15] Pires F. O., Dos Anjos C. A., Covolan R. J., Pinheiro F. A., St Clair Gibson A., Noakes T. D., Magalhaes F. H. and Ugrinowitsch C. Cerebral Regulation in Different Maximal Aerobic Exercise Modes. Front Physiol 2016; 7: 253 10.3389/fphys.2016.00253 27458381 PMC4932816

[16] Thomas K., Goodall S., Stone M., Howatson G., Gibson A. S. C. and Ansley L. Central and Peripheral Fatigue in Male Cyclists after 4-, 20-, and 40-km Time Trials. Medicine & Science in Sports & Exercise 2015; 47(3): 537–546 10.1249/mss.0000000000000448 25051388

[17] Abbiss C. R. and Laursen P. B. Describing and understanding pacing strategies during athletic competition. Sports Med 2008; 38(3): 239–252 10.2165/00007256-200838030-00004 18278984

[18] Enders H., V V. O. N. T. and Nigg B. M. Neuromuscular Strategies during Cycling at Different Muscular Demands. Med Sci Sports Exerc 2015; 47(7): 1450–1459 10.1249/MSS.0000000000000564 25380476

[19] Hounsgaard J., Hultborn H., Jespersen B. and Kiehn O. Intrinsic membrane properties causing a bistable behaviour of α-motoneurones. Experimental Brain Research 1984; 55(2): 391–394 10.1007/BF00237290 6086378

[20] Nehlig A., Daval J.-L. and Debry G. Caffeine and the central nervous system: mechanisms of action, biochemical, metabolic and psychostimulant effects. Brain Research Reviews 1992; 17(2): 139–170 10.1016/0165-0173(92)90012-b 1356551

[21] Gorassini M. A., Bennett D. J. and Yang J. F. Self-sustained firing of human motor units. Neuroscience Letters 1998; 247(1): 13–16 10.1016/s0304-3940(98)00277-8 9637398

[22] Johnson M. D. and Heckman C. J. Gain control mechanisms in spinal motoneurons. Frontiers in Neural Circuits 2014; 8: 81 10.3389/fncir.2014.00081 25120435 PMC4114207

[23] Walton C., Kalmar J. M. and Cafarelli E. Effect of caffeine on self-sustained firing in human motor units. The Journal of Physiology 2002; 545(2): 671–679 10.1113/jphysiol.2002.025064 12456842 PMC2290683

[24] Vestergaard M. and Berg R. W. Divisive Gain Modulation of Motoneurons by Inhibition Optimizes Muscular Control. The Journal of Neuroscience 2015; 35(8): 3711–3723 10.1523/JNEUROSCI.3899-14.2015 25716868 PMC6605555

[25] Shen J. G., Brooks M. B., Cincotta J. and Manjourides J. D. Establishing a relationship between the effect of caffeine and duration of endurance athletic time trial events: A systematic review and meta-analysis. J Sci Med Sport 2019; 22(2): 232–238 10.1016/j.jsams.2018.07.022 30170953

[26] De Pauw K., Roelands B., Cheung S. S., de Geus B., Rietjens G. and Meeusen R. Guidelines to classify subject groups in sport-science research. Int J Sports Physiol Perform 2013; 8(2): 111–122 10.1123/ijspp.8.2.111 23428482

[27] Goldstein E.R., Ziegenfuss T., Kalman D. et al International society of sports nutrition position stand: caffeine and performance. J Int Soc Sports Nutr 2010; 7(5). 10.1186/1550-2783-7-5 PMC2824625 20205813

[28] Gonçalves LS, Painelli VS, Yamaguchi G, et al Dispelling the myth that habitual caffeine consumption influences the performance response to acute caffeine supplementation. J Appl Physiol (1985). 2017;123(1):213‐220. 10.1152/japplphysiol.00260.2017 28495846

[29] Kirsch I. and Weixel L. J. Double-blind versus deceptive administration of a placebo.Behavioral Neuroscience 1988; 102(2): 5 10.1037/0735-7044.102.2.319. 3365327

[30] Saunders B., et al Placebo in sports nutrition: a proof‐of‐principle study involving caffeine supplementation. Scandinavian Journal of Medicine & Science in Sports 2017; 27(11): 1240–1247 10.1111/sms.12793 27882605

[31] Foad A. J., Beedie C. J. and Coleman D. A. Pharmacological and Psychological Effects of Caffeine Ingestion in 40-km Cycling Performance. Medicine & Science in Sports & Exercise 2008; 40(1): 158–165 10.1249/mss.0b013e3181593e02 18091009

[32] Peveler W. W. The Accuracy of Simulated Indoor Time Trials Utilizing a CompuTrainer and GPS Data. The Journal of Strength & Conditioning Research 2013; 27(10): 2823–2827 10.1519/JSC.0b013e318280ce76 23287833

[33] Sparks S. A., Williams E., Jones H., Bridge C., Marchant D. and McNaughton L. Test-retest reliability of a 16.1 km time trial in trained cyclists using the CompuTrainer ergometer. Journal Of Science And Cycling, 2016; 5(3), 35–41. 10.28985/jsc.v5i3.272

[34] Borg G. A. (1982). Psychophysical bases of perceived exertion. Med. Sci. Sports Exerc. 14, 377–381. 10.1249/00005768-198205000-00012 7154893

[35] Singh V. P. Entropy theory and its application in environmental and water engineering. 2013; New York: Wiley-Blackwell.

[36] Shannon C. E. A Mathematical Theory of Communication. Bell System Technical Journal 1948; 27(3): 379–423 10.1002/j.1538-7305.1948.tb01338.x 30854411

[37] Richman J. S. and Moorman J. R. Physiological time-series analysis using approximate entropy and sample entropy. Am J Physiol Heart Circ Physiol 2000; 278(6): H2039–2049 10.1152/ajpheart.2000.278.6.H2039 10843903

[38] Pincus S. M. Approximate entropy as a measure of system complexity. Proceedings of the National Academy of Sciences 1991; 88(6): 2297–2301 10.1073/pnas.88.6.2297 11607165 PMC51218

[39] Pethick J., Winter S. L. and Burnley M. Caffeine Ingestion Attenuates Fatigue-induced Loss of Muscle Torque Complexity. Medicine & Science in Sports & Exercise 2018; 50(2): 236–245 10.1249/mss.0000000000001441 28991045

[40] Lepers R., Maffiuletti N. A., Rochette L., Brugniaux J. and Millet G. Y. Neuromuscular fatigue during a long-duration cycling exercise. J Appl Physiol 2002 92(4): 1487–1493 10.1152/japplphysiol.00880.2001 11896014

[41] Tarnopolsky M. and Cupido C. Caffeine potentiates low frequency skeletal muscle force in habitual and nonhabitual caffeine consumers. Journal of Applied Physiology 2000; 89(5): 1719–1724 10.1152/jappl.2000.89.5.1719 11053318

[42] Kalmar J. M. The influence of caffeine on voluntary muscle activation. Med Sci Sports Exerc 2005; 37(12): 2113–2119 10.1249/01.mss.0000178219.18086.9e 16331138

[43] Meyers B. M. and Cafarelli E. Caffeine increases time to fatigue by maintaining force and not by altering firing rates during submaximal isometric contractions. J Appl Physiol 2005; 99(3): 1056–1063 10.1152/japplphysiol.00937.2004 15879163

[44] Stergiou N, Decker LM. Human movement variability, nonlinear dynamics, and pathology: is there a connection?. Hum Mov Sci. 2011;30(5):869‐888. 10.1016/j.humov.2011.06.002 21802756 PMC3183280

[45] Farina D., Negro F., Muceli S. and Enoka R. M. Principles of Motor Unit Physiology Evolve With Advances in Technology. Physiology 2016; 31(2): 83–94 10.1152/physiol.00040.2015 26889014

[46] Johnson M. D., Thompson C. K., Tysseling V. M., Powers R. K. and Heckman C. J. The potential for understanding the synaptic organization of human motor commands via the firing patterns of motoneurons. Journal of Neurophysiology 2017; 118(1): 520–531 10.1152/jn.00018.2017 28356467 PMC5511870

