# Correction: Caffeine increases motor output entropy and performance in 4 km cycling time trial

**DOI:** 10.1371/journal.pone.0246014

**Published:** 2021-01-22

**Authors:** Bruno Ferreira Viana, Gabriel S. Trajano, Carlos Ugrinowitsch, Flávio Oliveira Pires

The first and fourth author’s initials appear incorrectly in the citation. The correct citation is: Viana BF, Trajano GS, Ugrinowitsch C, Pires FO (2020) Caffeine increases motor output entropy and performance in 4 km cycling time trial. PLoS ONE 15(8): e0236592. https://doi.org/10.1371/journal.pone.0236592

The first author’s initials are also indexed incorrectly in PubMed. The correct initials are: Viana BF

The fourth author’s initials are also indexed incorrectly in PubMed. The correct initials are: Pires FO.
